# Ten Issues to Update in Nosocomial or Hospital-Acquired Pneumonia: An Expert Review

**DOI:** 10.3390/jcm12206526

**Published:** 2023-10-14

**Authors:** Francisco Javier Candel, Miguel Salavert, Angel Estella, Miquel Ferrer, Ricard Ferrer, Julio Javier Gamazo, Carolina García-Vidal, Juan González del Castillo, Víctor José González-Ramallo, Federico Gordo, Manuel Mirón-Rubio, Javier Pérez-Pallarés, Cristina Pitart, José Luís del Pozo, Paula Ramírez, Pedro Rascado, Soledad Reyes, Patricia Ruiz-Garbajosa, Borja Suberviola, Pablo Vidal, Rafael Zaragoza

**Affiliations:** 1 Clinical Microbiology and Infectious Diseases, Transplant Coordination, IdISSC & IML Health Research Institutes, Hospital Clínico Universitario San Carlos, 28040 Madrid, Spain; 2 Infectious Diseases Unit, La Fe (IIS) Health Research Institute, Hospital Universitario y Politécnico La Fe, 46026 València, Spain; 3 Intensive Medicine Service, Hospital Universitario de Jerez, 11407 Jerez, Spain; 4 Departamento de Medicina, INIBICA, Universidad de Cádiz, 11003 Cádiz, Spain; 5 UVIR, Servei de Pneumologia, Institut Clínic de Respiratori, Hospital Clínic de Barcelona, IDIBAPS, CibeRes (CB06/06/0028), Universitat de Barcelona, 08007 Barcelona, Spain; miferrer@clinic.cat; 6 Intensive Medicine Service, Hospital Universitario Valle de Hebrón, 08035 Barcelona, Spain; ricard.ferrer@vallhebron.cat; 7 Servicio de Urgencias, Hospital Universitario de Galdakao, 48960 Bilbao, Spain; juliojavier.gamazo@gmail.com; 8 Infectious Disease Service, Hospital Clinic, 08036 Barcelona, Spain; carolgv75@hotmail.com; 9 Emergency Medicine Service, Hospital Universitario San Carlos, 28040 Madrid, Spain; jgonzalezcast@gmail.com; 10 Unidad de Hospitalización a Domicilio, Hospital General Universitario Gregorio Marañón, 28007 Madrid, Spain; vgramallo@gmail.com; 11 Intensive Medicine Service, Hospital Universitario del Henares, 28822 Coslada, Spain; fgordo5@gmail.com; 12 Servicio de Hospitalización a Domicilio, Hospital Universitario de Torrejón, 28850 Torrejón de Ardoz, Spain; mmrubio@torrejonsalud.com; 13 Division of Respiratory Medicine, Hospital Universitario Santa Lucía, 30202 Cartagena, Spain; jvperpal@separ.es; 14 Department of Clinical Microbiology, ISGlobal, Hospital Clínic-University of Barcelona, CIBERINF, 08036 Barcelona, Spain; cpitart@clinic.cat; 15 Servicio de Enfermedades Infecciosas, Servicio de Microbiología, Clínica Universidad de Navarra, 31008 Pamplona, Spain; jdelpozo@unav.es; 16 Instituto de Investigación Sanitaria de Navarra (IdiSNA), 31008 Pamplona, Spain; 17 Intensive Medicine Service, Hospital Universitario y Politécnico La Fe, 46026 Valencia, Spain; ramirez_pau@gva.es; 18 Intensive Care Unit, Complejo Hospitalario Universitario Santiago de Compostela, 15706 Santiago de Compostela, Spain; pedrorascado@hotmail.com; 19 Neumology Department, Hospital Universitario y Politécnico La Fe, 46026 Valencia, Spain; solreyes07@gmail.com; 20 Microbiology Department, Hospital Universitario Ramón y Cajal, 28034 Madrid, Spain; pruizg@salud.madrid.org; 21 Intensive Medicine Service, Hospital Universitario Marqués de Valdecilla, Instituto de Investigación Sanitaria IDIVAL, 39011 Santander, Spain; borjasuberviola1977@gmail.com; 22 Intensive Medicine Service, Complexo Hospitalario Universitario de Ourense, 32005 Ourense, Spain; pablomopvc@hotmail.com; 23 Intensive Care Unit, Hospital Dr. Peset, 46017 Valencia, Spain; zaragoza_raf@gva.es

**Keywords:** nosocomial pneumonia, healthcare-associated pneumonia, etiology, epidemiology, diagnosis stewardship, radiologic findings, management, readmission, therapeutic failure, rescue, hospital at home, prevention, vaccination

## Abstract

Nosocomial pneumonia, or hospital-acquired pneumonia (HAP), and ventilator-associated pneumonia (VAP) are important health problems worldwide, with both being associated with substantial morbidity and mortality. HAP is currently the main cause of death from nosocomial infection in critically ill patients. Although guidelines for the approach to this infection model are widely implemented in international health systems and clinical teams, information continually emerges that generates debate or requires updating in its management. This scientific manuscript, written by a multidisciplinary team of specialists, reviews the most important issues in the approach to this important infectious respiratory syndrome, and it updates various topics, such as a renewed etiological perspective for updating the use of new molecular platforms or imaging techniques, including the microbiological diagnostic stewardship in different clinical settings and using appropriate rapid techniques on invasive respiratory specimens. It also reviews both Intensive Care Unit admission criteria and those of clinical stability to discharge, as well as those of therapeutic failure and rescue treatment options. An update on antibiotic therapy in the context of bacterial multiresistance, in aerosol inhaled treatment options, oxygen therapy, or ventilatory support, is presented. It also analyzes the out-of-hospital management of nosocomial pneumonia requiring complete antibiotic therapy externally on an outpatient basis, as well as the main factors for readmission and an approach to management in the emergency department. Finally, the main strategies for prevention and prophylactic measures, many of them still controversial, on fragile and vulnerable hosts are reviewed.

## 1. Introduction

Hospital-acquired pneumonia (HAP), or nosocomial pneumonia, is a pulmonary inflammatory process of infectious origin that is absent at the time of hospital admission; it develops after more than 48 h have elapsed and was not incubating at the admission time. Ventilator-associated pneumonia (VAP) is a significant sub-set of HAP. It appears in patients with an artificial airway more than 48 to 72 h after tracheal intubation [1,2,3]. VAP affects 10% to 20% of patients receiving mechanical ventilation for more than 48 h, and it represents more than 80% of pneumonias acquired in the intensive care unit (ICU). Both types of pneumonia are very relevant clinically [4], not only because of their high morbidity and mortality [5] (especially infections caused by multiresistant microorganisms) [6,7], but also because of their impact on quality of life, increased spending, and the high consumption of healthcare resources [8].

Many studies have found that HAP and VAP are associated with an increased risk of death. HAP is currently the main cause of death from nosocomial infection in critically ill patients, with an incidence of 5 to 10 cases per 1000 hospital admissions; by contrast, VAP affects approximately 10–25% of all patients in ICUs. The estimated mortality rate of HAP is 20–30%, but it is higher (20–50%) in VAP [9,10].

The management of HAP and VAP requires an interprofessional team consisting of specialists in infectious diseases, pulmonary diseases, intensive care, anesthesiologists, microbiologists, and other healthcare professionals such as nurses and pharmacists [11]. Without adequate treatment, morbidity and mortality in these processes will remain high [12].

Although the guidelines for the approach to this type of infection are internationally implemented in all health systems, there is variability in the diagnostic–therapeutic management, with differences in morbidity and mortality rates, the achievement of microbiological diagnosis, the request for complementary studies, the choice of antimicrobial regimen in a scenario of multidrug resistance, or the diversity of care applied. In addition, information is continually emerging that generates debate or requires an update in its management.

The aim of the present paper was to review the 10 topics that have undergone the greatest update in nosocomial and healthcare-associated pneumonia. Topics such as the renewal of the etiological perspective through the use of new syndromic platforms, the implementation of diagnostic lung ultrasound, the criteria for ICU admission or clinical stability, and a therapeutic update in the context of bacterial multidrug resistance, including continuity of care at home, causes of therapeutic failure, and rescue options, among others, were chosen. The main prevention strategies and prophylactic measures, many of them still controversial, in fragile and vulnerable hosts were also reviewed.

## 2. Material and Methods

Design. From the Study Group of Infection in the Critically Ill Patient of the Spanish Society of Infectious Diseases and Clinical Microbiology (GEIPC-SEIMC), experts were requested in January 2023 from all scientific societies attending to nosocomial and healthcare-associated pneumonia (listed in this document’s affiliation), grouping two authors per topic. They were asked for a narrative review. Search strategy. Between January and June 2023, the experts performed a bibliographic search of their corresponding topics in PubMed (http://www.ncbi.nlm.nih.gov/pubmed/, accessed on 26 January 2023), Embase (http://www.elsevier.com/online-tools/embase/, accessed on 26 January 2023) and Scopus (http://www.elsevier.com/online-tools/scopus), choosing those that, in their experience, were most relevant or most current, up to a maximum number of 15 references, excluding the rest. Drafting. In June, the texts were received from the experts, with a limit of two pages per topic. Some of them, due to the content of the assigned topic, were also asked to include a figure or a table. Between June and July, the coordinators integrated the texts. Revision. Between July and August, all the experts had the opportunity to read the complete text and raise objections and changes.

## 3. Results

### 3.1. Etiologic Update of Hospital-Acquired Pneumonia

Nosocomial pneumonia (NP) is defined as pneumonia that has developed in that have been patients admitted to the hospital for >48 h and is caused by pathogens that are present in hospital settings. Within NP, ventilator-associated pneumonia (VAP) develops in intensive care unit (ICU) patients who have been mechanically ventilated for at least 48 h. On the other hand, healthcare-associated pneumonia (HAP) has been proposed as a separate category from community-acquired pneumonia (CAP) as it develops in patients who are not hospitalized but that have risk factors for being colonized by multidrug-resistant (MDR) microorganisms. The risk factors defining the HAP population were identified as nursing home or extended care facility, recent hospitalization, dialysis, and chronic wound care [8].

According to data published in the latest report of the Survey on the Prevalence of Healthcare-associated Infections and Antimicrobial Use in Acute Care Hospitals in Spain (EPINE-2022) [13], *Pseudomonas aeruginosa* (18.2%) and *Staphylococcus aureus* (12.2%) were the main pathogens causing HAP, followed by *Klebsiella pneumoniae* (6.9%) and *Escherichia coli* (6.7%). These data are similar to those described in other studies conducted in other geographical areas, although frequencies can vary among regions. For example, a multicenter study in the United States involving 17,819 patients with NP, VAP, and NP that required subsequent mechanical ventilation found that *S. aureus* was the most frequently isolated organisms, occurring in nearly 40% of each pneumonia group. *P. aeruginosa* was the second most prevalent pathogen and was isolated between 16 and 19% in the different groups. Finally, *E. coli* and *K. pneumoniae* accounted for 12–13% of all infections, except for a lower prevalence of *E. coli* in VAP patients (8.7%) [14]. On the other hand, in Spain, according to data from the National Surveillance Study of Nosocomial Infection in Intensive Medicine Services (ENVIN-HELICS 2022) [15], VAP accounted for 35% of the infections acquired in the ICU. Again, the three most frequent bacterial pathogens were *P. aeruginosa* (17.5%), *S. aureus* (12.1%), and *K. pneumoniae* (10.3%), followed by *E. coli* (7.5%), *Enterobacter cloacae* (7.3%), and *Serratia marcenscens* (7%). Finally, among the bacterial pathogens, other non-fermenting Gram-negative bacilli such as *Stenotrophomonas maltophilia* and *Acinetobacter baumannii* are particularly relevant in ICU patients, causing 5.5% and 0.7%, respectively, of VAP according to data from the ENVIN-HELICS study [15]. It should be noted that in other geographical areas, such as Eastern European countries, the frequency of *A. baumannii* can be as high as 20% [16].

Some studies have found similarities between the etiology of CAP and HAP and a variability in the proportion of multidrug-resistant pathogens causing HAP episodes [17]. Nevertheless, in the current epidemiological scenario, a substantial proportion of NP and HAP episodes are caused by MDR microorganisms, and local epidemiology will largely determine the most frequently isolated resistant pathogens in an area. A recent study monitoring the antimicrobial resistance (SENTRY Antimicrobial Surveillance Program) of microorganisms isolated from respiratory samples of patients with HAP in the USA and Europe found wide variations in antimicrobial resistance [16]. According to data from this study, the percentage of methicillin resistance in *S. aureus* in Western European countries was 16% in 2019, which is close to the 14% reported in the ENVIN study among *S. aureus* causing VAP. In contrast, these percentages are much higher in Eastern Europe and the USA, with 38.6% and 40.1%, respectively. Among *Enterobacterales* the presence of carbapenemases was mainly associated with *K. pneumoniae*. The highest percentages of carbapenemase-producing strains in 2019 were detected in Eastern Europe (23.6%), while in Western Europe and the USA these percentages were significantly lower (1.4% and 1.7%, respectively). Regarding the type of carbapenemase, the KPC class predominated in Western Europe and the USA, while OXA-48 and MBLs were the most common carbapenemases found in Eastern Europe. Overall, ceftazidime/avibactam were highly active (>90% susceptibility) against *E. coli* and *K. pneumoniae* isolates (excluding MBL-producing strains) from all geographic regions. Among non-fermenting bacilli, *P. aeruginosa* resistance to meropenem was significantly higher in Eastern Europe (51.7%) than in other regions, where resistance rates were around 20%. Overall, ceftolozane/tazobactam and ceftazidime/avibactam were highly active (>95% susceptibility), although in Eastern Europe activity decreased to 80%. Finally, in *A. baumannii*, resistance to meropenem was around 30% in the USA and Western Europe, while in Eastern Europe, values above 90% were described in 2019 [16].

The use of new diagnostic molecular techniques has led to an increased interest in the role of respiratory viruses as potential etiological agents of pneumonia. NV and HAP can also have a viral etiology, with SARS-CoV-2, influenza, respiratory syncytial virus, and rhinovirus accounting for most cases [18]. In mechanically ventilated patients, viruses belonging to the *Herpesviridae* family, namely herpes simplex virus (HSV) and cytomegalovirus, can be reactivated and cause bronchoneumonitis or VAP [18].

Although empirical antibiotic treatment must be appropriate and administered as soon as possible, a rapid microbiological diagnostic test should be performed in a timely fashion to establish the etiological diagnosis [19]. Gram stain and sputum culture, blood culture, urinary antigens, PCR for MRSA in pharyngeal swab, and culture of bronchoscopy samples, if possible, are recommended by the international guidelines [1]. Molecular tests have been developed to simultaneously detect and quantify multiple respiratory pathogens, as well as some genes related to antimicrobial resistance [20]. Several commercial platforms are currently available for comprehensive molecular testing for respiratory pathogens that cause pneumonia (respiratory viruses, bacteria, and fungi) and for the main resistance genes of the most common bacteria causing pneumonia. Such nucleic-acid detection methods include PCR or reverse-transcription PCR and microarray-based assays and can establish a microbial diagnostic in few hours [21,22,23]. Examples of integrated molecular systems used in pneumonia diagnosis include: GeneXpert (Cepheid, Sunnyvale, CA, USA), which is used for the identification of *S. aureus* and its methicillin resistance in less than one hour, leading to a rapid treatment optimization; and Filmarray (bioMérieux, Marcy-l’Étoile, France) or STAT (Qiagen, Hilden, Germany), which include multiple target pathogens and resistance markers. Like all microbiological techniques, molecular methods are not without limitations. On one hand, the sensitivity and specificity of molecular techniques vary widely, and they do not provide information about the viability of the identified microorganisms. Resistance gene detection may lead to a discrepancy between genotype and phenotype, and other obstacles include the emergence of new resistance mechanisms not included in this panels, which can lead to false negatives, as well as the detection of genotypic resistance that may not necessarily indicate clinically significant resistance. The use of this rapid molecular diagnostic test presents challenges in routine implementation and must be supplemented with standard culture producers.

Table 1 summarizes the main techniques for rapid diagnosis of HAP that have a time to response of two hours or less [24,25,26,27]. The combination of new and conventional techniques will enable the more precise detection of pathogens causing pneumonia. These new techniques should not be considered to be a replacement for conventional methods but rather a complement that will enhance the management of pneumonia patients and reduce the emergence of antibiotic resistance.

To evaluate the implementation of new molecular techniques in a clinical microbiology laboratory, studies assessing the impact of these assays on patient outcomes and their cost-effectiveness are necessary. Clinical trials assessing their impact on patient prognosis are scarce. In the MAGIC-BULLET study, Filmarray^®^ showed a sensitivity of 78.6%, a specificity of 98.1%, a positive predictive value of 78.6%, and a negative predictive value of 96.6% in respiratory samples. Furthermore, Filmarray^®^ provided results within only one hour directly from respiratory samples with minimal sample processing time [28]. Moreover, the BioFireFilmArray-Pneumonia plus Panel was evaluated in 79 patients with pneumonia in the intensive care unit (ICU). The implementation of a syndromic pneumonia panel improved time to diagnosis, identified new pathogens not detected by cultures in 49% of the cases, and resulted in a significant reduction in antibiotic consumption. The study also demonstrated the positive value of PCR syndromic testing in the management of pneumonia in ICUs with high rates of MDR/XDR nosocomial pathogens [29].

Regarding cost-effectiveness studies of molecular tests, Leone et al. assessed the economic impact using Cepheid Xpert real-time PCR for the rapid diagnosis of MRSA in respiratory samples (BAL and miniBAl) from patients suspected of VAP [30]. They considered two possibilities for empirical antibiotic treatment: a more expensive one (150 euros/day for patients with renal failure) and a less expensive one (150 euros/day). The cost of the test was 45 euros. The authors demonstrated that, in the case of the empirical treatment costing 150 euros/day, the test was cost-effective regardless of the prevalence of MRSA, and, in the case of the treatment costing 50 euros/day, the test was cost-effective if the prevalence was >25%.

### 3.2. Importance of Respiratory Sample Quality for the Diagnosis of Ventilator-Associated Pneumonia

The lack of a reference standard for the diagnosis of ventilator-associated pneumonia has led to the coexistence of different diagnostic strategies in the collection of respiratory samples for microbiological study. The main clinical trials that analyzed the prognostic impact of these strategies obtained disparate results explained by differences in their methodological design, which, for example, excluded immunocompromised patients, patients with chronic diseases, patients treated with quinolones or carbapenems, or patients colonized by resistant bacteria [31,32]. More than two decades later, the main scientific societies in Europe and the USA apply different approaches. The American Thoracic Society/Infectious Diseases Society of America (ATS/IDSA) guidelines suggest the use of endotracheal aspirate samples [1], while the International ERS/ESICM/ESCMID/ALAT [8] recommends invasive diagnosis with bronchoscopy, valuing the higher quality of the samples and the potential benefits in reducing antibiotic exposure and its impact on antibiotic resistance. In addition, the incidence of ventilator-associated pneumonia in epidemiological registries is affected by the quality of the respiratory specimen selected for diagnosis. The national registry of infection in the critically ill patient in Spain (ENVIN HELICS) contemplates this discrimination.

The clinical strategy based on obtaining upper respiratory tract samples by tracheal aspiration is less invasive, safer, easier to perform, inexpensive, and does not require specialized personnel. The invasive strategy is based on obtaining samples from the lower respiratory tract through fiberoptic bronchoscopy, mostly obtained by bronchoalveolar lavage, which collects material from the alveolar space after instilling a quantity of sterile liquid. Bronchial brushing or biopsies are bronchoscopic techniques less used in ventilated patients. A great advantage is the direct view of the bronchial tree, which allows us, in addition to sampling, to evaluate the state of the bronchial mucosa, assess lesions, and to detect bleeding or lesions of a non-infectious nature. In expert hands, it is a safe procedure with hardly any complications [33,34], reinforces the clinician’s confidence in the use of antibiotics, and reduces overdiagnosis of pneumonia by avoiding misinterpretation of proximal airway colonization. From the microbiological point of view, in the absence of previous antimicrobial therapy, it has a high negative predictive value to consider other foci in the suspicion of infectious complications, and the sample is of greater value and allows a more accurate diagnosis that includes non-culture-dependent techniques (galactomannan, beta-D-glucan, etc.). In an intermediate point between both strategies is bronchoalveolar mini-lavage, whose main disadvantage is that it is a blind technique in which the area where the sample is to be taken cannot be directed [35]. Regarding predictive values of different respiratory samples for ventilator-associated pneumonia diagnosis, A Conway Morris et al. [36] documented that tracheal aspirate over diagnosed VAP compared with BAL, obtaining a low positive predictive value of qualitative (21%) and quantitative cultures (30%). Mini-BAL seemed to have more accuracy for diagnosis due to the high contamination risk in tracheal aspirate [35], and it might be a reasonable alternative when bronchoscopy is not available [37]. Table 2 shows the advantages and disadvantages of both diagnostic strategies.

### 3.3. Implementation of Imaging Techniques (CT and Lung Ultrasound) in Diagnosis of NP-HAP

NP-HAP presents a high morbi-mortality during hospitalization. In addition, it is the cause of more serious infections, and multiresistant microorganisms (MDR) are frequently involved in etiology. An accurate diagnosis is essential to establish early treatment and to reduce mortality.

The role of the image is key in NP-HAP. Chest X-ray (CXR) is the gold standard in the initial evaluation of pneumonia. The guidelines recommend it when pneumonia is suspected [1,27]. However, the performance of computed tomography scans (CT) has increased in recent years. In clinical or epidemiological contexts, CT scans have been useful to differentiate among bacterial, viral, or fungal origin. In addition, it allows a better representation of the pattern, distribution of pneumonia, and severity. It plays an important role in the diagnosis of complications and the treatment of MDR pneumonia.

In NN-HAP, CXR may have low diagnostic sensitivity. The main reasons: bedridden patients, exacerbated comorbidities, greater severity, weak patients, and admitted to the ICU. In these patients, it is a challenge to obtain quality images, and a CXR can be negative for diagnosis. Likewise, in nosocomial infections, symptoms can develop very early, and the abnormality may not be visible on the CXR, especially in immunocompromised patients [38]. In NP-HAP, CT scans have greater diagnostic sensitivity. They can provide early identification of radiological signs of pneumonia in seriously ill and immunocompromised patients. CT scans can show findings suggestive of pneumonia up to 5 days before conventional radiography. This is important to establish early treatment and reduce mortality. Recently, studies have been published that demonstrate the superiority of CT scans versus CXR in the diagnosis of pneumonia [39,40,41]. Miyashita et al. demonstrated the superiority of CT scan compared to CXR in NP-HAP, with statistically significant differences. Furthermore, the low diagnostic accuracy of the CXR was correlated with the deterioration of the patient’s functional status [42].

The role of the CT scans is not only limited to the detection of NP-HAP, but also helps the clinician in making the etiological differential diagnosis. Some imaging findings and pattern identification may help suggest etiologies. In NP-HAP, the most frequent germs involved are Gram negative, *Enterobacteriaceae* species, Gram positive cocci, and fungal infections, and they are often MDR. However, there are limitations when making a radiological-only etiological diagnosis. NP-HAP frequently involves several microorganisms, and radiographic patterns vary due to pre-existing or coexisting lung diseases. In addition, a microorganism can produce different radiological patterns, and, very importantly, they can change according to the immunological status of the patient. Thus, in *Aspergillus fumigatus* infection, the CT scan is the diagnostic test of choice. Typical signs are single or multiple pulmonary nodules with the halo or cavitation sign, which may not be present in immunocompromised patients. In these patients, the radiographic findings may be irregular and non-specific consolidations, ground glass patterns, and no evidence of nodules [43,44]. Pulmonary nodules can also be seen in bacterial infections, mycobacterial infections, or *Nocardia* spp. However, in NP-HAP, patchy bronchopneumonia is the most common finding, which is usually caused by Gram negatives. Viral pneumonia may be described as ground glass or pulmonary interstitial infiltrates.

CT scans allow us to assess the severity of pneumonia, and they better detail the involvement and lung extension. In a study carried out in patients with severe pneumonia in the ICU, CT scans was shown to be superior to CXR in demonstrating lung involvement, pleural effusion, and atelectasis. This involved making changes in clinical procedures and treatment. In addition, the affectation of the number of lobes was correlated with the lowest PO2/FiO2 and, consequently, severity. On the other hand, other studies did not show the superiority of CT scans over CXR in terms of severity. Clinical outcomes, including length of stay, ICU admission, mechanical ventilation, and mortality, were similar in both groups [45,46]. In conclusion, the CT scan has shown superiority in many studies for a more accurate and earlier diagnosis in patients with NP-HAP, improving the prognosis of these patients. However, it is more expensive, produces greater irradiation, and, sometimes, due to the clinical instability of some patients, it cannot be used. From the future perspective, lung ultrasound (LUS) could be a cost-effective, easy-to-use, and safe alternative in NP-HAP, particularly in resource-limited settings.

Diagnosis of pneumonia is primarily based on clinical signs and symptoms and imaging tests like CXR or CT scans. However, these tests may not always be readily available, are associated with radiation exposure, and may not provide a real-time evaluation of disease progression. LUS has emerged as an alternative diagnostic tool for the detection of pneumonia, providing real-time imaging and having no radiation exposure.

Several studies have evaluated the accuracy of LUS in the diagnosis of pneumonia. A systematic review and meta-analysis by Llamas-Alvarez et al. [47] included 16 studies with a total of 2378 patients and reported a sensitivity of 94% and specificity of 96% for the diagnosis of pneumonia. Similarly, Xia et al. [48], conducted a meta-analysis of 11 studies, including 1093 patients, and reported a pooled sensitivity of 93% and specificity of 95%. Liu et al. [49] conducted a prospective study of 133 patients with community-acquired pneumonia (CAP) and reported that LUS had a sensitivity of 96.8% and specificity of 96.6% in the diagnosis of CAP.

Several studies have compared the diagnostic accuracy of LUS with CXR and CT scans. Nazerian et al. [50] conducted a prospective study of 161 patients with pulmonary consolidations and reported that LUS had a sensitivity of 98% and specificity of 94% for the diagnosis of consolidations compared to CT scans. Amatya et al. [51] conducted a study in a low-resource setting and reported that lung ultrasound had a sensitivity of 92% and specificity of 78% for the diagnosis of pneumonia compared to CXR. Ellington et al. [52] conducted a prospective study of 219 children with radiographically confirmed pneumonia and reported that LUS had a sensitivity of 98.4% and specificity of 100%.

The OCTOPLUS study [53] is a multicenter randomized controlled trial that aims to evaluate the diagnostic accuracy of LUS and low-dose CT scans for the diagnosis of pneumonia in elderly patients. The study will enroll 876 patients aged 65 years or older and compare the diagnostic accuracy of lung ultrasound and low-dose CT scans with standard of care strategies.

In conclusions, LUS has emerged as a promising diagnostic tool for the diagnosis of NP-HAP, providing real-time imaging and having no radiation exposure. Several studies have reported high sensitivity and specificity for the diagnosis of pneumonia using LUS. Furthermore, lung ultrasound has been shown to have better diagnostic accuracy than CXR and comparable diagnostic accuracy to CT scans. The OCTOPLUS [53] study aims to evaluate the diagnostic accuracy of lung ultrasound and low-dose CT scans for the diagnosis of pneumonia in elderly patients and may provide further evidence on the utility of LUS in this patient population.

There are some limitations to consider. Firstly, while LUS is highly accurate in diagnosing pneumonia, it may not be able to identify the specific cause of the pneumonia. This information may be important for determining the most effective treatment. Secondly, LUS requires specialized training and equipment, which may not be available in all settings or from all practitioners. This can limit the widespread use of this diagnostic tool. Thirdly, LUS may be limited by the patient’s body habitus or the presence of underlying lung disease, which can make it difficult to obtain clear and accurate images. Lastly, while LUS is non-invasive and does not expose patients to ionizing radiation, it may not be appropriate for patients with certain medical conditions or those who are unable to tolerate the positioning required for the exam. Overall, while LUS is a valuable diagnostic tool for pneumonia, it is important to consider these limitations and ensure that it is used appropriately and in conjunction with other diagnostic methods when necessary.

### 3.4. Update on Antimicrobial Treatment in HAP-NP and VAP-New Evidence

In the case of a patient with suspected NP, antibiotic treatment must be appropriate and administered as early as possible. However, delayed appropriate empirical therapy is a persisting and frequent practice [8,54]. In a recent analysis with 56,357 patients with GNB infections, delayed appropriate therapy was received by 2800 (46.2%) patients with resistant and 16,585 (33.0%) patients with susceptible infections [55]. The authors showed, using multivariate analysis, that delayed appropriate therapy was associated with worse outcomes, including a ~70% increase in LOS, a ~65% increase in total in-hospital costs, and a ~20% increase in the risk of in-hospital mortality/discharge to hospice, regardless of susceptibility status. Similar results have been found in a meth analysis that includes different types of bacterial infections with very consistent results [56].

The inclusion in our therapeutic arsenal of new antibiotics and the incorporation in our daily practice of microbiological rapid diagnostic tests give us the opportunity to overcome these problems. For this reason, both entities have become the cornerstone of the approach to the treatment of nosocomial pneumonia in our centers [57].

New diagnostic tests have been developed, such as multiplex polymerase chain reaction (MPCR), exhalome analysis, and chromogenic tests [58]. MPCR has reported sensitivity of 89.2% and specificity of 97.1% using BAL samples, and 71.8% sensitivity and 96.6% (range, 95.4–97.5%) using endotracheal aspirates (ETA) [59].

The development of new antibiotics, such as Ceftolozane/tazobactam (CFT-TAZ), Ceftazidime/avibactam (CAZ/AVI), Meropenem/Vaborbactam (MERO/VAR), Imipenem/Relebactam (IMI/REL), and cefiderocol (CEF) has broadened the treatment options for patients with suspected MDRO Gram negative infection. All of them offer some advantages: apart from demonstrated efficacy in clinical trials for approval, they present a better in vitro activity, less resistance, and can also be used within the scope of an antibiotic policy aimed to reserve (spare) carbapenem [9,19,57]. In fact, Spanish and European recent guidelines have included them as first lines or alternative therapies [60,61]. However, many of them have yet to define their place within the treatment of microorganisms with high resistance through clinical studies.

CFT/TAZ presents greater in vitro activity against *P. aeruginosa* with less resistance than the remaining current anti-pseudomonal agents in global terms [62]. CFT/TAZ also exhibits the lowest mutant prevention concentration (MPC) against *P. aeruginosa*, as well as colistin and quinolones (2 mg/L) [63]. The clinical trial ASPECT-NP [64] revealed a favorable result for patients who suffer from HAP that require invasive MV treated with CFT/TAZ (mortality at 28 days 24.2% vs. 37%) and also in those patients in whom initial antibiotic treatment failed (mortality at 28 days: 22.6% vs. 45%). In patients with bacteraemia, a trend towards a higher rate of clinical cure (10.5% vs. 36%), without statistical significance, was observed in CFT/TAZ treated patients. In this clinical trial, higher levels of microbiological cure in pneumonia caused by *P. aeruginosa* was also observed in patients who received CFT/TAZ.

On the other hand, CAZ/AVI was associated with better survival rates in patients with bacteraemia who required rescue treatment in infections caused by KPC-producing *Enterobacteriaceae* [65]. When the strain is an OXA producer, the treatment of choice is CAZ-AVI [66,67]. KPC producing strains can be treated with CAZ-AVI [66,67], MER-VAB [68], IMI-REL [69], or cefiderocol [70]. None of these antibiotics are free of the risk of resistance [69,70]. Strains resistant to ceftazidime-avibactam because of the onset of mutant KPC can be treated with MER-VAB or IMI-REL [71]. In case of resistance to CAZ-AVI and/or MER-VAB, IMI-REL has shown adequate in vitro activity [72]. Moreover, the addition of relebactam significantly improves the activity of imipenem against most species of *Enterobacteriaceae* (lowering the minimum inhibitory concentration (MIC) by 2- to 128-fold) depending on the presence or absence of β-lactamase enzymes. Against *P. aeruginosa*, the addition of relebactam also improves the activity of imipenem (MIC reduced eightfold). IMI-REL may be useful in patients with suspected or documented P. aeruginosa infections [69]. The great activity of relebactam against KPC-2 and KPC-3 β-lactamase may confer certain advantages on IMI-REL in treating these strains [73].

In the MERINO trial [74], randomized patients hospitalized with bacteraemia caused by enterobacteria resistant to ceftriaxone received antibiotic treatment with meropenem or piperacillin/tazobactam. The clinical outcomes were unfavorable for the group of patients that received piperacillin/tazobactam, which cuts down the treatment options for these infections. In published clinical trials, both CFT/TAZ or CAZ/AVI [64,75] antibiotics demonstrated appropriate activity and clinical efficacy to ESBL-E (extended spectrum beta-lactamase-producing enterobacteria), whereby they arise as new alternatives and may be included in carbapenem-spare regimens.

Cefiderocol may be considered a good candidate to treat these infections due to its excellent in vitro activity against all classes of beta-lactamase-producing Gram negatives (including carbapenemase class A, B, and D producers), as well as against non-fermenting Gram negatives such as *P. aeruginosa*, *Acinetobacter* spp. and *S. maltophilia* [76]. MBL producing strains resistant to aztreonam should be treated with cefiderocol [77]. Whilst aztreonam-avibactam is not available, good results have been reported using CAZ-AVI combined with aztreonam [78]. The combination of CAZ/AVI and ATM is considered synergistic, and it is an effective therapeutic option, particularly against *Klebsiella* spp. species and *E. coli* isolates producing more than one carbapenemase gene of metallo β-lactamases and serine β-lactamases. However, the in vivo efficacy and safety of this regimen have to be evaluated. To the best of our knowledge, there was only one recent study, conducted by Falcone et al. [79], to clinically compare the effect of the CAZ/AVI and ATM combination to other active antibiotics on the outcome of patients with bloodstream infections due to MBL-producing Enterobacterales. It showed that the treatment with CAZ/AVI and ATM was linked with lesser clinical failure at day 14, lower mortality at day 30, and shorter length of hospitalization.

In the CREDIBLE-CR study [70], although the clinical cure of patients with pneumonia and bacteremia treated with cefiderocol versus best available therapy was similar in both treatment groups, crude all-cause mortality at 14, 28, and 49 days was higher in patients treated with cefiderocol. This difference in mortality was observed mainly in patients with *A. baumannii* infections. On the other hand, different real-life cases have been published in which cefiderocol has shown excellent results in complex infections produced by carbapenem-resistant, extremely resistant, and pan-resistant *A. baumannii*. An observational study including 124 patients with CR-AB infections compared cefiderocol-and colistin-containing regimens [80]. Thirty-day mortality in patients receiving colistin-compared to those who received cefiderocol-containing regimens was 55.8% versus 34% (*p* = 0.018). In a multivariable analysis, cefiderocol therapy was protective with 30-day mortality, and nephrotoxicity was more common in the colistin group. Cefiderocol should be considered as a therapeutic option against *A. baumanni* in patients with severe infections, especially when the unique alternative available was colistin. Another issue not resolved is whether it should be used in monotherapy or as combination therapy.

**Figure 1:**
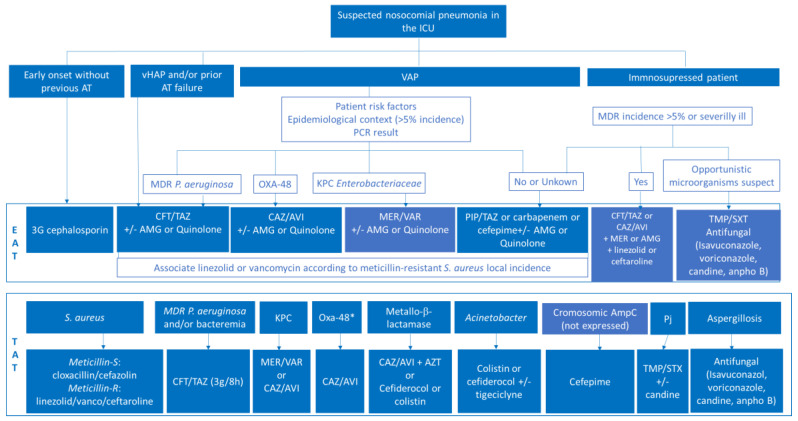
Modified PANNUCI algorithm from empirical to targeted treatment on nosocomial pneumonia in ICUs in European countries (both immunocompetent and immunosuppressed). AT: antimicrobial therapy; vHAP: ventilated hospital-acquired pneumonia; VAP: ventilator-associated pneumonia; MDR: multidrug resistant; PCR: polimerasa chain reaction; CFT/TAZ: ceftolozan/tazobactam; CAZ/AVI: ceftazidime/avibactam; PIP/TAZ: piperacillin/tazobactam; AMG: aminoglycoside; AZT: Aztreonam; EAT: empirical antimicrobial treatment; TAT: targeted treatment; OXA-48: OXA-48 Carbapenemase; KPC: *Klebsiella pneumonie* Carbapenemase; MER-VAR: MEROPENEM-VABORBACTAM; IMI-REL: IMIPENEM-RELEBACTAM; ESBL-E: extended spectrum beta-lactamase-producing enterobacteria; PJ: *Pneumocystis jiroveccii.* * If Oxa-48 susceptible to CAZ/AVI.

An algorithm that includes the priorities analyzed to update empirical and targeted treatment in critically ill patients has been designed (Figure 1). Following the prior PANNUCI algorithm [19] after analyzing the onset, the previous use of antimicrobials, or clinical condition (vHAP or VAP), empirical antimicrobial therapy has been chosen based on risk factors, previous colonization, local flora, and/or use of rapid techniques. Therefore, targeted therapy is selected depending on type of microorganism isolated and the possible advantages of one antimicrobial over others.

### 3.5. Nebulized Treatment in NP-HAP

Global emergence of multidrug-resistant (MDR) and extensive drug-resistant (XDR) Gram negative bacteria (GNB) has increased the risk of treatment failure, especially for hospital-acquired (or healthcare-associated) or ventilator-associated pneumonia (HAP/VAP).

Nebulized administration enables delivery of high doses of antibiotics directly to the lungs. Antibiotic nebulization provides high intrapulmonary concentrations, pronouncedly higher than the minimum inhibitory concentration of causative pathogens of lung infection and higher than the minimal concentrations preventing resistant emergence, with low systemic passage and resulting side effects. It represents a promising approach to optimize the treatment of HAP/VAP due to MDR and XDR GNB while limiting systemic antibiotic exposure. Aminoglycosides and colistin (colistimethate sodium or methanesulfonate) are the most common nebulized antibiotics.

Even though 2017 ESCMID practice guidelines reported safety concerns and weak evidence of benefit supporting the use of aerosolized antibiotics in mechanically ventilated patients [81], many intensive care unit (ICU) practitioners use aerosolized antibiotics, as shown in an international survey on this topic [82]. The administration of inhaled antibiotics in ICU patients remains, however, controversial, notwithstanding extensive pre-clinical and clinical research and potential indications associated with the emergence of bacterial antibiotic resistances [83].

The efficacy and safety of adjunctive inhaled antibiotic therapy for VAP was updated in a systematic review and meta-analysis of RCTs, published in 2021 [84]. The outcomes assessed were clinical cure, microbiological eradication, mortality, and adverse events. Eleven RCTs and 1210 patients were included in this analysis. Compared with the use of IV injection alone, the use of adjunctive inhaled antibiotic therapy improved the rates of clinical cure (relative risk (RR) 1.13, 95% CI [1.02,1.26]) and microbiological eradication (RR 1.45, 95% CI [1.19,1.76]) in VAP patients. However, despite these improvements, mortality was not reduced (RR 1.00, 95% CI [0.82,1.21]). Adjunctive antibiotics delivered through the respiratory tract were not associated with a higher risk of renal impairment but were associated with an increased risk of bronchospasm (RR 2.74, 95% CI [1.31,5.73] during treatment.

Therefore, the systematic use of nebulized antibiotics is not supported by the currently available evidence as a routine therapeutic strategy for VAP. In ICUs, nebulized antibiotics may be considered for treatment of VAP caused by resistant pathogens in patients at high risk of therapeutic failure or as a last resort in case of uncontrolled infection with IV antibiotics [85].

Prophylactic administration of nebulized antibiotics to prevent VAP has yielded encouraging results. Compared with placebo or no treatment, nebulized antibiotics reduced the incidence of VAP (odds ratio [OR]: 0.46; 95% confidence interval [CI]: [0.22–0.97) without any effect on ICU mortality (OR: 0.89; 95% CI: 0.64–1.25) or occurrence of VAP due to MDR pathogens (OR: 0.67; 95% CI: 0.17–2.62) in a meta-analysis including six comparative trials and involving 1,158 patients [83]. This approach is, however, not yet recommended, and large randomized controlled trials (RCTs) should be conducted to confirm its benefit and explore the impact on antibiotic selection pressure.

Although the optimal nebulized drug dosing regimen is not clearly established, high doses of antibiotics are required to reach the infected lung parenchyma. Breath synchronized nebulizers do not allow delivery of high doses. Vibrating-mesh nebulizers perform better than jet nebulizers. Epithelial lining fluid concentrations largely overestimate lung interstitial space fluid concentrations in patients receiving nebulized antibiotics [86]. To optimize lung deposition of nebulized antibiotic in VAP, specific ventilator settings should be used during nebulization to reduce inspiratory flow velocity; humidification and warming of inspired gas should be interrupted to avoid a rainout effect in the circuits and airways, and sedation should be administered to avoid dys-synchrony with the ventilator [87]. As nebulized aminoglycosides and colistin broadly diffuse in the systemic circulation of patients with extensive bronchopneumonia, monitoring of plasma trough concentrations is recommended during the period of nebulization.

An expert opinion review article proposed that future RCTs should compare a 3–5 day nebulization of amikacin or colistin to a 7-day intravenous administration of a new cephalosporine/ß-lactamase inhibitor. Inclusion criteria should be a VAP or ventilator-associated tracheobronchitis caused by documented XDR or pan drug resistant GNB [88]. The dose of nebulized antibiotics should be titrated according to the level of drug resistance of causative pathogens, but always at much higher doses than those recommended for IV administration [89]. The expected benefits from nebulized antibiotics are a shorter time to clinical cure, a decrease in antibiotic-induced renal toxicity, a shorter duration of IV antibiotic administration, and a reduction in mechanical ventilation duration in patients with VAP [90,91,92].

Beyond the advances in the design of future RCTs, other aspects that should draw our attention in this field in the near future are the development of new high-performance devices that would enhance lung deposition and novel inhaled anti-infectious therapies. On the first path, we want to highlight intratracheal spray of antibiotics and high-performance mesh nebulizers. On the second path, various drugs are under development to strengthen the anti-infectious therapeutic arsenal, like bacteriophages and immunomodulatory drugs [85].

In conclusion, inhaled antibiotics are a common therapeutic practice used in mechanically ventilated patients across ICUs. Strong evidence of their benefits still needs to be produced through well-conducted RCTs considering the specificity of nebulization during mechanical ventilation. Current use should be evaluated on a case-by-case basis among patients with MDR or XDR VAP. Translation of positive preclinical studies into clinical implementation is complex.

### 3.6. Approach to the Management of NP-HAP in the Immunosuppressed Patient

Immunosuppression is reported to be associated with higher rates of infection related to reduced defense mechanisms and higher exposure to healthcare facilities and antimicrobial courses. Pneumonia, mainly nosocomial or hospital-acquired or healthcare-associated pneumonia (NP/HAP), is the leading infection. A global incidence of pneumonia of 52.2 episodes per 100 allo-HSCT/year has been reported, and up to 30% of pulmonary transplant recipients suffer from pneumonia [92,93]. The consequences of NN/HAP are clearly depicted in the described associated mortality, 46.3% in allo-HSCT and 50–70% in solid organ recipients [92,93]. Appropriate diagnosis and treatment are the key elements to improve patient outcomes.

Nosocomial healthcare-associated pneumonia diagnosis. The first challenge lies in the frequency of non-infectious differential diagnoses, including pulmonary toxicities of oncological treatments, acute pulmonary oedema, intra-alveolar hemorrhage, or lesions related to the underlying disease itself. Differential diagnosis may be difficult due to atypical presentations in immunocompromised patients, including the absence of inflammatory syndrome.

Immunosuppressed patients with nosocomial pneumonia should undergo a chest computed tomography (CT) scan in order to detect less evident lesions or infiltrates and to better localize them. Although radiological patterns may suggest certain etiologies (consolidations in bacterial etiologies, ground-glass opacities in viral pneumonia, or specific signs in invasive pulmonary aspergillosis), atypical presentations are frequent, and imaging should not be a surrogate for microbiological diagnosis [94]. Other strategies to document differential diagnoses, such as cardiac and pleural ultrasound, have also emerged as primary diagnostic tools in this population.

The use of biomarker tests, such as (procalcitonin) PCT or (c-reactive protein) CRP, for diagnosing and monitoring pneumonia has been suggested, although most studies have not been specifically conducted in immunocompromised individuals.

Microbiological documentation is the cornerstone of the diagnosis of pneumonia in the immunocompromised patient. It must first be noted that the type of immunosuppression is an important element to take into account in the etiological assessment. Biological samples and microbiological tests are depicted in Table 3. Respiratory samples are obviously the most important specimens. The diagnostic yield of sputum samples in this setting is usually very poor, especially due to poor-quality samples. Fiberoptic bronchoscopy (FOB) with bronchoalveolar lavage (BAL) remains, to this day, the most exhaustive technique. FOB allows the possibility of making macroscopic findings, such as herpetic or fungal lesions. BAL has been shown to improve the rate of the etiological diagnosis of severely immunosuppressed patients, leading to a change in their therapeutic management and an improvement in outcomes [95,96,97]. BAL viral and fungal PCR assessments are well-established diagnostic techniques. More recently, bacterial tests (simplex or multiple-PCR tests) are increasing their diagnostic performance, particularly in patients previously exposed to antibiotics. Strålin et al. reported that BAL culture was positive in 5/24 (21%) cases, while this rate reached 14/24 (58%) cases for PCR. The interest in PCR also lies in its capacity to provide a rapid etiological diagnosis coupled with the possibility of detecting the presence of mechanisms of antimicrobial resistance [98]. In some selected patients with difficulties in diagnosis and/or unfavorable evolution, it may be necessary to perform transbronchial lung biopsy or open lung biopsy.

Administering an adequate empiric antibiotic treatment should be the most important goal for physicians. In this scenario, the empiric antibiotic treatment approach for NP-HAP should include coverage for *Pseudomonas aeruginosa*, other Gram negative bacilli (GNB), and Gram positive cocci, especially *S. aureus* [92]. Currently, the high prevalence of multidrug-resistant bacteria worldwide represents a major challenge for physicians. The knowledge of the local distribution of pathogens associated with NP-HAP and their antimicrobial susceptibilities should be the first step in deciding the best therapeutic decisions.

The last guideline for adults with HAP/VAP [1] has recommended the use of the same empirical antibiotics over the past 25 years (ceftazidime, cefepime, piperacillin-tazobactam, meropenem, amikacin, vancomycin, and linezolid). However, it is concerning that these antibiotics are not still valid for a high percentage of GNB isolated in several hospitals, and new options to treat MDR-GNB and MRSA NP-HAP are now available. A current approach might be to ensure double coverage of *P. aeruginosa* infection with a high dose of antipseudomonal antibiotics including an active beta-lactam. Starting with an antibiotic bolus and follow up with extended perfusions will be recommended. The most suitable antibiotic nowadays will be ceftolozane/tazobactam 3 g/8 h or ceftazidime/avibactam 2.5 g/8 h plus meropenem 2 g/8 h or amikacin 20–30 mg/kg per day, all IV. Gram positive coverage should include the use of linezolid 600 mg/8–12 h or ceftarolina 600 mg/8 h iv. Empiric antibiotic therapy should be replaced as soon as the microbiological cultures are known, adjusting the treatment to the results obtained. If an infection by opportunistic microorganisms is suspected, treatment with trimethoprim-sulfamethoxazole (15–20 mg/kg/6–8 h of trimethoprim) or isavuconazol 200 mg/8 h within the first 48 h followed by a regimen each 24 h should be considered for *Pneumocystis jirovecii* and *Aspergillus* spp. coverage. Other antifungals with activity against *Aspergillus* spp. that may eventually be used are voriconazole (6 mg/kg BD the first day followed by 4 mg/kg BD thereafter) or liposomal amphotericin B (3–5 mg/kg/day). A section on therapeutic recommendations in immunosuppressed patients is included in the algorithm for therapeutic recommendations (Figure 1).

In immunosuppressed patients, the wide variety of possible nosocomial pneumonia etiologies requires the systematic performance of multiple microbiological tests. Empirical treatment should be broad to cover the majority of possible etiologies and should include the treatment of opportunistic microorganisms in case of clinical suspicion.

### 3.7. Management of Nosocomial Pneumonia and Health-Associated Pneumonia at Home

NP and HAP are traditionally considered to be heterogeneous entities, but they share a higher morbidity and mortality than community-acquired pneumonia. One of the causes of this greater severity lies in the more frequent involvement of multidrug-resistant microorganisms in their etiology [99]. These pathogens usually require antimicrobial treatments that are more difficult to administer on an outpatient basis, either intravenously or orally. These two circumstances, together with their lower incidence, mean that the experience of their treatment outside the hospital is limited, and no specific series have been found in the literature. Unlike community pneumonias [100], American, European, and Spanish guidelines and recent reviews do not consider this possibility [101,102].

Within the NP we can distinguish three entities with some differences in etiology, diagnosis, treatment, and prognosis. Two of them, ventilator-associated pneumonia (VAP) and nosocomial ventilator-associated pneumonia (vHAP), rarely require treatment outside the hospital. Nosocomial pneumonia not requiring ventilation (nvHAP), despite its theoretical lower severity, is a cause of prolonged hospital stay and increased morbidity and mortality in patients admitted to medical and surgical services [103] and a higher risk of hospital readmission after discharge [14].

A type of pneumonia halfway between NP and CAP is that of pneumonia acquired in closed non-hospital institutions where elderly and/or physically and/or cognitively challenged individuals usually reside (nursing home acquired pneumonia). These pneumonias also have a higher mortality than CAP, and multidrug-resistant microorganisms play an important role in their etiology [104]. Given the fragility of the patient and the availability of health resources in these centers, there is experience available on the treatment of pneumonia in these centers without hospital admission [105], including an Australian series in which treatment is provided by a Hospital at Home service [106].

Hospital at Home (HaH) is a modality of care that allows patients, who would otherwise have to receive care in a hospital, to be cared for in their usual place of residence (their home or a social-health center). HaH has proven to be an effective and safe alternative in several infectious diseases, among which community-acquired pneumonia is one of the most experienced [107]. The procedure most frequently performed by HaH services is outpatient intravenous antimicrobial therapy. It is also the one for which cost savings compared to conventional hospitalization have been most reliably demonstrated [108]. It is important to remark that HaH not only provides the infusion of antimicrobials but also ensures close monitoring of the patient based on home visits, telephone calls, point of care devices, and telemonitoring tools.

As we have already pointed out, the experience of NP treatment in HaH is scarce and generally not specified in general series. In a study published by our group 5 years ago of nosocomial-acquired infections included in a multicenter database treated in thirty Spanish HaH units [109], 16% of the 9314 episodes included were due to nosocomial-acquired infections. The 184 NPs were the fourth cause of admission (13%) in HaH for hospital-acquired infections after urinary tract (28%), skin (20%), and intra-abdominal (17%) infections, while, in community-acquired infections, respiratory infections constituted the second group only after urinary tract infections.

Unlike other nosocomial infections, such as urinary tract infections, catheter-related bacteremia, or surgical wound infection, the etiology of NP in non-ventilated patients is quite often unknown, requiring broad-spectrum antimicrobial therapy covering both Gram positive, including methicillin-resistant *S. aureus*, and Gram negative, including *Pseudomonas* spp. and ESBL-producing enterobacteria. While this broadening of the spectrum may pose an added difficulty for home antimicrobial therapy, there are several technological and organizational resources that allow HaH services to administer these more complex regimens.

For a decade, there has been published experience of HaH treatment of infections by multidrug-resistant microorganisms [110]. In this study, intensive use is made of self-administration and elastomeric devices, but it is possible to administer many of the antimicrobials used then and those marketed since then with electronic infusion pumps and with one or two daily nursing visits, taking into account the stability of the antimicrobial once reconstituted [111], the availability of nursing resources, and the degree of collaboration of the patient and caregiver (Table 4). If the agent is stable at room temperature, it is possible to use both elastomeric devices and electronic pumps for continuous or extended infusions, which have demonstrated their efficacy and safety in Gram negative NP [112].

### 3.8. Management of Healthcare-Associated Pneumonia Presenting and Attending at the Emergency Department

Lower respiratory tract infections are a common reason for emergency department (ED) consultations, with pneumonia accounting for approximately 1.35% of patients attended [113]. Pneumonia is also the leading cause of sepsis, septic shock, death, and admission to the ICU [114].

In addition, the population attending the ED is typically older, frail, with accumulated comorbidity, or institutionalized; pneumonia is, in fact, one of the most common infections arising amongst nursing home residents. Healthcare contact, instrumentation (cure of ulcers, vascular or vesical catheters, hemodialysis, feeding tubes), immunosuppressive treatments, and antibiotic pressure are some of the factors associated with MDRO infections [113,114,115] and also criteria for healthcare-associated pneumonia (HCAP) [116].

HCAP can be defined as the one that occurs in patients who meet one of the following criteria: (a) hospitalization for 2 days or more in the preceding 90 days; (b) residence in a nursing home or extended care facility; (c) home infusion therapy (including antibiotics); (d) chronic dialysis within 30 days; (e) home wound care; or (f) family member with a multidrug-resistant pathogen. It has been documented that up to 50% of patients with pneumonia attending the ED may meet the criteria for HCAP, and only 10% to 30% of these patients have infections caused by resistant bacteria. The use of such criteria with high sensitivity and limited specificity may lead to excessive utilization of broad-spectrum antimicrobials, unnecessary costs, and an increased prevalence of resistant bacteria. Finally, the concept of HCAP does not take into account the severity of the disease, and it is well known that resistant bacteria appear most frequently in patients with severe disease. In conclusion, though widely employed and specifically identified in many guidelines, the HCAP concept lacks the necessary precision to identify a profile of patients at risk of infection by resistant organisms, and, therefore, an etiologic approach is recommended by many authors based on individual risk factors for infection caused by resistant organisms and severity of disease.

On the other hand, broad-spectrum empirical antimicrobial treatment for HCAP, which included methicillin-resistant *Staphylococcus aureus* (MRSA) and double anti-pseudomonal therapy, as suggested in 2005 in the American Thoracic Society (ATS)/Infectious Diseases Society of America (IDSA) guidelines [116], has not been shown to improve survival or decrease hospital stay [117,118]. In fact, with this approach, a third of patients may be overtreated [119], resulting in increased antibiotic resistance, worse outcomes, and higher costs. Accordingly, the most recent update of the IDSA/ATS guidelines made a change in therapy approach, suggesting that each hospital creates antibiograms to aid professionals in selecting the best antibiotics and reduce the unnecessary use of dual Gram negative and empiric MRSA treatments [1].

Antibiotic treatment decisions during the treatment of patients at the ED are typically empirical, based on results of previous cultures and knowledge of the local epidemiology and the evaluation of the risk factors for MDRO, with adequate selection of the antimicrobial treatment being a challenge. The consequences of an inappropriate selection of antibiotic treatment can be critical, particularly for the most severe patients, where it can have a great impact on their prognosis [120].

Several scores have been developed to identify patients at higher risk of MDRO infections, with the DRIP score demonstrating good predictive value in a pneumonia study cohort, with the potential to decrease antibiotic overutilization [121]. At a threshold of >4 points, the DRIP score demonstrated a sensitivity of 0.82 (95% CI, 0.67 to 0.88), a specificity of 0.81 (95% CI, 0.73 to 0.87), a positive predictive value (PPV) of 0.68 (95% CI, 0.56 to 0.78), and a negative predictive value (NPV) of 0.90 (95% CI, 0.81 to 0.93).

Another score, developed in a cohort study comprising 54 Spanish EDs, demonstrated effective prediction of MDRO infection risk [122]. Although the study did not specifically focus on pneumonia patients, the scoring system achieved an AUROC of 0.79 (95% CI, 0.75–0.83) in the derivation cohort and 0.76 (95% CI, 0.70–0.82) in the validation cohort. This system categorizes patients into six groups based on their score, which corresponds to the probability of contracting an MDRO infection.

Early screening for MDRO infection is particularly crucial for patients with severity criteria upon admission to the ED (respiratory failure or need for mechanical ventilation, sepsis, or septic shock), where the impact of an inadequate selection of antibiotic treatment on prognosis is greater [119,123]. The application of scoring indices originally designed for community-acquired pneumonia (CAP) can also be effective for HCAP. However, it is important to note that the predictive ability of each pneumonia severity score for mortality was found to be less accurate for HCAP when compared to CAP. Among these scores, the pneumonia severity index (PSI) was found to be the most useful in predicting mortality in HCAP [124].

Baseline biological samples should be collected in the ED for microbiological analysis to obtain an etiological diagnosis and antimicrobial sensitivity, which may allow for de-escalation of therapy. However, conventional microbiological tests have limited sensitivity and specificity to diagnose HCAP, and it usually takes several days to know the final antimicrobial susceptibility profile [125]. Thus, the ability to rapidly identify bacteria and the presence of antimicrobial resistance is important in this era of antimicrobial resistance. Diagnostic panels for pneumonia that offer more sensitive and faster results than conventional techniques could be of immense value [126].

In summary, the best strategies for clinical management of HCAP at the ED include the use of predictive scores to identify the risk of MDRO infection, appropriate assessment of pneumonia severity, implementation of de-escalation strategies, and careful individual assessment of comorbidity and functional status.

### 3.9. Therapeutic Failure and Rescue in HAP-NP

Therapeutic failure in HAP-NP. The clinical and microbiologic response of a NP-HAP can be assessed easily from 72 h after starting antibiotic treatment. Suspicion of therapeutic failure is based on several factors: (a) Persistence of clinical symptoms, (b) radiographic progression, (c) impairment of organ failure or appearance of a new organ failure, (d) no decreased in biomarkers, and (e) isolation of a new pathogen on day 3. However, persistence of the original pathogen does not seem to be associated with a worse prognosis [127]. Among the clinical markers of treatment failure, physicians should consider persistence of fever, no improvement in the ratio of arterial oxygen partial pressure to fractional inspired oxygen (PaO_2_/FiO_2_), persistence of purulent respiratory secretions, and new-onset septic shock or multiple-organ dysfunction syndrome. The choice of an inappropriate antibiotic treatment, which is generally related to the existence of uncovered multidrug-resistant organisms (MDROs), is probably the most important risk factor for therapeutic failure. Moreover, sub-therapeutic antibiotic concentrations can be also suspected. In critically ill patients, multiple underlying derangements provoke pathophysiological alterations that change the PK/PD of drugs and therefore provoke reduced drug concentration at the infectious focus. Measuring blood antibiotic concentrations is recommended to optimize antimicrobial administration. Higher than licensed dosing regimens of β-lactams is safe and effective in reducing the rate of therapeutic failure, especially in critically ill patients with augmented renal clearance [122,128]. Table 5 shows the different causes that should be considered in patients with therapeutic failure. Bronchoscopic evaluation is recommended for patients with therapeutic failure to rule out the presence of MDROs, *Legionella* spp., opportunistic pathogens including fungi (specially *Aspergillus* spp.), and viruses.

Rescue in HAP-NP. It is essential to follow the recommended guidelines for the treatment of patients with NN-HAP. Inappropriate empirical antimicrobial therapy is a significant issue in the treatment of infections caused by multidrug-resistant organisms (MDRO), and the choice of an inappropriate antibiotic treatment is a crucial prognostic factor that is potentially modifiable. Studies have shown that inappropriate antibiotic therapy is associated with an increased risk of mortality, especially in patients with severe infections, and this risk is even higher in patients with MDRO infections [129,130,131]. According to Zaragoza et al. [19], even with combination therapy there is a high likelihood of receiving inadequate empirical treatment for MDRO infection. The study analyzed data from the National Surveillance Programme of Intensive Care Unit (ICU)-Acquired Infection in Europe Link for Infection Control through Surveillance (ENVIN-HELICS) [132] and found that approximately 30% of patients were at risk of receiving ineffective treatment.

To improve patient outcomes, it is important to promptly adjust or modify antibiotic therapy in patients with documented or suspected MDRO infections, especially if the clinical course is not adequate. To accurately make a decision of change, it is imperative to focus the analysis on patients who have received an inappropriate empirical treatment. We should be able to identify patient and epidemiological-disease-related factors linked to an adverse outcome to propose an antimicrobial salvage therapy to avoid a failure scenario [133]. In a study conducted by Esperatti et al. [134], they confirmed a correlation between several clinical variables recorded between 72 and 96 h after initiating treatment and the prognosis of 335 patients with NP-HAP. The study found that patients with a worse clinical outcome had the absence of improved oxygenation, the need for mechanical ventilation, persistence of fever or hypothermia together with purulent respiratory secretions, radiological worsening in more than 50% of the lung area, or the development of septic shock or multi-organ failure following the onset of antibiotic treatment. These factors can aid in monitoring the progress of patients with NP-HAP and help healthcare professionals make informed decisions about salvage treatment options. MDR *P. aeruginosa*, ESBL-E, MRSA, *A. baumannii*, and CPE are the most encountered MDROs in NP-HAP. Knowledge of the local prevalence of each MDRO is essential to guide an effective treatment. Ceftolozane/tazobactam (CFT/TAZ) and ceftazidime/avibactam (CAZ/AVI) are new antibiotics that offer benefits such as superior in vitro activity, less resistance, and demonstrated efficacy in clinical trials for approval. They can be administered as part of an antibiotic policy designed to preserve carbapenems. The choice of antibiotics should consider the site of infection, clinical severity, and presence of risk factors for MDRO acquisition, comorbidities, and existing MDROs in each unit/hospital [9,134]. Data extracted from an in vitro study suggest that CAZ/AVI plus aztreonam could be an option to treat infections caused by metallo-β-lactamase producing *Enterobacteriaceae* [135]. Cefiderocol has been granted approval by the US Food and Drug Administration for treating NP-HAP, as well as infections caused by Gram negative pathogens that are carbapenem-resistant, such as *Acinetobacter* spp. In the case of NP-HAP, colistin is not an effective drug to consider unless it is administered through aerosolization. Prolonged courses of antimicrobial therapy promote more resistance. European guidelines recommend antibiotic treatment for NP-HAP for no longer than 7 days [8]. However, the duration of therapy for MDRO infections is not clearly established. The use of rapid diagnostic tests, such as multiplex polymerase chain reaction, exhalome analysis, and chromogenic tests, is expected to revolutionize the diagnosis and treatment of NP-HAP. Early and appropriate antibiotic salvage treatment, including new ones, is crucial to improving NP-HAP outcomes due to the severity of patients. The goal is to improve inefficacious treatments, adjust the spectrum and duration of treatment, and minimize potential adverse effects and interactions.

### 3.10. Measures for Prevention and Prophylaxis of HAP-NN: Controversies to Be Resolved

The main pathogenic mechanism of NP (associated or not with mechanical ventilation) is micro-aspiration of bacteria from the oropharyngeal or gastrointestinal tract. Therefore, those situations that favor the passage of these bacteria or limit the main defense mechanism against them (coughing) will increase the risk of pneumonia. Mechanical ventilation is the most important risk factor because it combines both conditions: the orotracheal tube prevents closure of the glottis, and the low level of consciousness (due to the underlying pathology or the sedation that frequently accompanies mechanical ventilation) limits the patient’s ability to cough.

Some measures have been proposed to prevent VAP:

- Semi-recumbent position (30–45%). Compared with supine position, it seems that the semi-recumbent position reduces VAP incidence, mainly in patients receiving enteral nutrition [136,137]. The evidence is limited, and other positions have been proposed, such as lateral Trendelemburg, which has shown efficacy but increased adverse events [138].
- Strict hand hygiene before and after handling the airway and single-use sterile gloves. Hand washing with alcohol-based solutions should be performed before and after manipulating the airway. The use of gloves does not prevent hand hygiene. Although usually included in bundles, the application of hand hygiene programs have been shown to reduce the incidence of VAP by themselves [139,140].
- Education and training of all staff involved in airway management
- Encourage early extubation. Duration of mechanical ventilation is one of the main risk factors for VAP. Application of weaning protocols and daily extubation trials have shown a reduction in VAP incidence [141]. The use of non-invasive ventilation in weaning from mechanical ventilation shortens mechanical ventilation time and, therefore, reduces the risk of VAP [142].
- Continuous monitoring of endotracheal cuff pressure. Automatic monitoring and adjustment of endotracheal cuff pressure have been shown to reduce micro-aspirations [143]; however, this measure has failed to demonstrate a reduction in the incidence of VAP in clinical trials [144,145].
- Continuous aspiration of subglottic secretions. The most common route by which bacteria reach the lower respiratory tract is through aspiration of accumulated secretions above the cuff of the endotracheal tube. Continuous or intermittent aspiration of subglottic secretions has been proposed as a way to reduce the incidence of VAP. Clinical trials have consistently shown a reduction in the incidence of VAP related with subglottic aspiration [146,147] and even a reduction in mortality [148].
- No scheduled ventilator circuit changes, unless soiled or malfunctioning. Ventilator circuits should be changed only if visibly soiled or malfunctioning (or following the manufacturer’s instructions). Scheduled changes do not reduce the incidence of VAP and increase healthcare costs [149].
- Administration of antibiotics for 24 h after intubation of comatose patients. Several cohort studies and randomized trials have shown a significant reduction in the incidence of early-onset VAP through the use of systemic antibiotics in the first 24 h after intubation [150,151]. The effect of this measure, by itself, seems to be restricted to the reduction in early-onset VAP in comatose patients, without affecting mortality or duration of mechanical ventilation but decreasing ICU length of stay [152].
- Oral care with clorhexidine 0.12–0.2%. The effect of oral care with chlorhexidine is controversial. It has been related to a reduction in VAP incidence following cardiac surgery [153], while other studies have shown no benefits in non-cardiac surgery patients, and it may be even harmful in some patients [137,154,155,156]. Oral care with chlorhexidine is not recommended by the SHEA/IDSA/APIC guidelines [157].
- Selective digestive decontamination (SDD). The goal of SDD is to reduce the incidence of VAP caused by endogenous microorganisms. SDD includes the administration of systemic antibiotics and the administration of non-absorbable topical antibiotics in the oropharynx (oral paste) and through the nasogastric tube (solution). The most frequently used combination is polymyxin E, tobramycin, and amphotericin B. Dozens of clinical trials have shown a significant reduction in the incidence of VAP without adverse effects (mainly related to the appearance of antimicrobial resistance) [157]. Recent meta-analyses found that the reduction in the incidence of VAP is accompanied by a significant decrease in mortality when complete digestive decontamination is applied (oropharyngeal, digestive, and short systemic antibiotic therapy [158,159,160].

As we have mentioned, numerous measures have been shown to reduce nosocomial pneumonia and VAP incidence [161]. The combined application of all of them in the form of bundles seems to maximize their effectiveness [162]. A national program in Spanish ICUs, with an intervention that consisted of implementing a VAP prevention bundle that included the measures described above, reduced the incidence of VAP by 50% [163].

## 4. Conclusions

Optimal treatment of HAP requires an interprofessional team composed of multiple specialists to ensure current, homogeneous, effective, and safe treatment. New molecular diagnostic techniques have made it possible to identify viruses, fungi, and other opportunistic microorganisms, along with the usual bacteria, as causative agents of pneumonia in both immunocompetent and immunosuppressed patients, improving the prognosis in the most severe patients and facilitating the initiation of appropriate treatment. In regard to imaging techniques, CT lung scans better represent specific patterns and help suspicion of certain etiologies, while ultrasound techniques allow the image to be viewed in real-time without irradiating the patient. Modern antimicrobial treatments marketed in the last five years are adjusted to the multidrug-resistant genotype of the etiologic agent, some of them with a spectrum capable of covering more than one of these genotypes. The therapeutic ceiling of nebulized antibiotics is not yet defined. It is increasingly necessary to include continuity of care in the therapeutic strategy of these patients, and the defervescence period can be completed in the home hospitalization unit, since the galenic formulation of many antibiotics allows it. Poor control of the focus and sub-inhibitory concentrations of the antimicrobials used in the infection are the most frequent causes of therapeutic failure. Anticolonization measures and those that avoid micro-aspiration reduce the incidence of pneumonia, especially in those who require ventilatory support.

## Figures and Tables

**Table 1:** Molecular techniques for rapid diagnosis of HAP that have a time to response of two hours or less.

Molecular Technique	Methodology	Target	Time to Response
VERIGENE^®^ Respiratory Pathogens Flex Test (RP Flex) (Luminex)	Multiplex RT-PCR/Solid-phase microarray with gold nanoparticles	Inf (A, H1, H3, H1 2009, B), AdV, VRS (A, B), MpV, PiV (1, 2, 3, 4), RnV, BPer, BPar, BHol	2 h
Film Array Respiratory 2 plus Panel (bioMerieux, Marcy-l’Étoile, France)	Nested multiplex RT-PCR/Melting analysis	Inf (A, H1, H3, H1 2009, B), VRS, AdV, CoV (229E, OC43, NL63, HKU1, MERS), MpV, PiV (1, 2, 3, 4), RnV/EV, BPer, BPar, MPne, CPne	45 min
BiofireFilmArray Pneumonia Plus Panel (bioMerieux, Marcy-l’Étoile, France)	Nested multiplex RT-PCR/Melting analysis	ABau, EClo, ECol, HInf, KAer, KOxy, KPne, MCat, Prot, PAer, SMar, SAur, SAga, SPne, SPyo, CPne, LPne, MPne, Inf (A, B), VRS, AdV, CoV, MERS, MpV, PiV, RnV/EV, mecA, mecC, MERJ, KPC, NDM, OXA48, VIM, IMP, CTXM	1 h 15 min
Xpert^®^ XpressFlu/RSV (Cepheid, Sunnyvale, CA, USA)	Real-time RT-PCR	Inf (A, B), VRS	20 min
QIAstat-Dx Respiratory SARS-CoV- 2 Panel (QIAGEN, Hilden, Germany)	Real-time RT-PCR	Inf (A, H1, H1 2009, H3, B), VRS (A, B), AdV, CoV (229E, OC43, NL63, HKU1), SARS-CoV-2, MpV, PiV (1, 2, 3, 4), RnV/EV, BPer, MPne, CPne, LPne	1 h 10 min
cobas^®^ Liat^®^ (Roche, Basel, Switzerland)	Real-time PCR	Inf (A, B), VRS	20 min
ePlexRespiratory Pathogen (RP) Panel (GenMark, Carlsbad, CA, USA)	Electrowetting/Microarray Solid phase/Detection electrochemistry	Inf (A, H1, H3, H1 2009, B), VRS (A, B), AdV, PiV (1, 2, 3, 4), MpV, CoV (229E, OC43, NL63, HKU1), RnV, MPne, CPne	1 h 30 min

Abau: Acinetobacter calcoaceticus baumannii complex; AdV: Adenovirus; CoV: Coronavirus; CPne: Chlamydophila pneumoniae; CxV: Coxsackie virus; DPO: dual priming oligonucleotide; EClo: Enterobacter cloacae complex; ECol: *Escherichia coli*; EV: Enterovirus; HInf: Haemophilus influenzae; Inf: Influenza; H1: Influenza A H1N1 epidemic; H1 2009: Influenza A H1N1 pandemic; KAer: Klebsiella aerogenes; KOxy: Klebsiella oxytoca; KPneu: Klebsiella pneumoniae; MCat: Moraxella catharralis; MERS: Middle East Respiratory Syndrome; MPne: Mycoplasma pneumoniae; MpV: Metapneumovirus; PAer: *Pseudomonas aeruginosa*; PiV: Parainfluenza virus; Prot: *Proteus* spp.; RnV: Rhinovirus; RT-PCR: reverse transcription polymerase chain reaction; SAga: *Streptococcus agalactiae*; SAur: *Staphylococcus aureus*; SMar: Serratia marcescens; SMal: Stenotrophomonas maltophilia; SPne: Streptococcus pneumoniae; SPyo: Streptococcus pyogenes; RSV: Sincitial Respiratori Virus.

**Table 2:** Strategies for diagnosis of ventilator-associated pneumonia.

	Non-Invasive Strategy Tracheal Aspiration	Invasive Strategy Bronchoscopy and Bronchoalveolar Samples
Advantages	Quick Easy to perform Safe Inexpensive	Lower respiratory tract guided sample obtained High specificity Distinguish between infection and colonization Noninfectious diagnosis by direct visualization Safe
Disadvantages	Upper respiratory tract Difficult to differentiate from colonization Risk of overuse of antibiotics	Need for trained staff

**Table 3:** Microbiological procedures to consider in NP/HAP diagnosis in immunosuppressed patients.

	Technique	Microorganisms	Advantages	Disadvantages
Respiratory sample (BAL)	Gram stain	Bacteria, yeast	Immediate results	False negatives. Observer dependent
	Ziehl-Nielsen stain, modified Ziehl-Nielsen stain	*Mycobacterium tuberculosis*, *non-tuberculous mycobacteria, Nocardia* spp.		
	Fungal morphology (KOH, calcofluor, papanicolau, H&E, GMS or PAS staining, ink staining)	Fungus		
	Culture	Bacteria, fungus, virus		Time dependent. False negatives
	Galactomannan (ELISA, lateral-flow)	*Aspergillus* spp.	Immediate results	Discrepancy among techniques
	Direct fluorescent antibodies	*Aspergillus* spp. *Mycobacterium tuberculosis*		
	PCR (simplex or multiple)	*Aspergillus* spp. *Pneumocystis jirovecii, Mycobacteria*, Virus (respiratory virus, CMV, VHS), Bacteria	Immediate results. High sensitivity	Positivity does not always imply infection. Microorganisms not included in multiple test
Nasopharyngeal swab	PCR (simplex or multiple)	Mainly respiratory virus	Immediate results	Positivity does not always imply infection
Serum sample	Galactomannan	*Aspergillus* spp.	Rapid results	False negative in non-neutropenic patients
	(1-3)-β_D-glucan	Fungus (except mucorales and *Crypctococcus* spp.)	High negative predictive value. Treatment evaluation	False negatives
	Cryptococcal antigen			
Urine sample	Soluble antigen tests	*Histoplasma* spp., *Cryptococcus*, *S. pneumoniae*, *L. pneumophila*	Immediate results	
Blood	Culture	Bacteria, fungus		Time dependent. False negatives.
	PCR (simplex or multiple)	CMV, VHS, VEB, Adenovirus. Bacteria	Rapid results	Microorganisms not included in multiple test

**Table 4:** Antimicrobials frequently used for nosocomial pneumonia or healthcare-associated pneumonia in the home setting.

Antibiotic	Standard Dose	Microbiological Target	Stability at 25 °C	Home iv Infusion Device/Modality	
				Electronic Pump	Elastomeric Pump/Gravity
Piperacillin-tazobactam	4/0.5 g every 6–8 h	*Pseudomonas aeruginosa, Enterobacteriaceae*	>24 h	Yes	Optional (self-administration) ^b^
Ceftazidime	1–2 g every 8 h	*Pseudomonas aeruginosa, Enterobacteriaceae*	>24 h	Yes	Optional (self-administration) ^b^
Cefepime	2 g every 8–12 h	*Pseudomonas aeruginosa, Enterobacteriaceae*	>24 h	Yes	Optional (self-administration) ^b^
Meropenem	1–2 g every 8 h	*Pseudomonas aeruginosa, Enterobacteriaceae ESBL*	<24 h	No ^a^	Yes (self-administration) ^c^
Ertapenem	1 g every 24 h	*Enterobacteriaceae ESBL*	<24 h	No needed	
Ceftolozane-tazobactam	2/1 g every 8 h	*Pseudomonas aeruginosa*	Up to 24 h	Yes	Optional (self-administration) ^b^
Ceftazidime-avibactam	2/0.5 g every 8 h	*Pseudomonas aeruginosa, other resistant Enterobacteriaceae*	<24 h	No ^a^	Yes (self-administration)
Amikacin *	15–20 mg/kg/d	*Pseudomonas aeruginosa*	>24 h	No needed	Yes
Tobramycin *	5–7 mg/kg/d	*Pseudomonas aeruginosa*	>24 h	No needed	Yes
Gentamicin *	5–7 mg/kg/d	*Pseudomonas aeruginosa*	>24 h	No needed	Yes
Aztreonam *	1–2 g every 8 h	*Pseudomonas aeruginosa, Enterobacteriaceae*	>24 h	Yes	Optional (self-administration) ^b^
Levofloxacin *	500 mg every 24 h	*Pseudomonas aeruginosa, Enterobacteriaceae*	>24 h	No needed	Gravity (presentation as 100 mL ready-to-use containers)
Linezolid	600 mg every 12 h	MRSA	>24 h	No	Gravity (presentation as 300 mL ready-to-use containers)
Vancomycin	15–20 mg/kg every 12 h	MRSA	>24 h	Yes	Consider two nursing visits. Optional: self-administration ^b,d^
Ceftaroline	600 mg every 12 h	MRSA	Up to 24 h (6 mg/mL in sodium chloride 0.9%, protected from light)	Yes	Consider two nursing visits. Optional: self-administration ^b^
Ceftobiprole	500 mg every 8 h	MRSA	Up to 24 h (2 mg/mL in sodium chloride 0.9%, protected from light)	Yes	Gravity (2-h infusion)

(* in combination); MRSA: methicillin-resistant Staphylococcus aureus. ESBL: Extended spectrum beta-lactamase. ^a^ Unless the dilution is kept refrigerated. ^b^ For selected patients and only after a thorough training process, self-administration may be offered as an alternative to electronic pump infusion. ^c^ Some practitioners combine two nursing visits with the use of the electronic pumps to avoid self-administration. ^d^ At least 1 h infusion.

**Table 5:** Causes of therapeutic failure in patients with NP-HAP. PK/PD: pharmacokinetics/pharmacodynamics.

Cause	Recommendation
Inadequate antibiotic treatment	Escalate based on microbiological results.
Sub-therapeutic antibiotic concentrations	Increase antimicrobial dosing. Use extended or continuous antibiotic infusions to optimize PK/PD parameters
New pathogens isolated	Antimicrobial treatment according to microbiological data
Undrained pyogenic focus (i.e., empyema)	Therapeutic drainage
Drug fever	Change antibiotic treatment
A non-infectious illness presenting as NP-HAP	Management as appropriate
